# A Novel Patent Ductus Arteriosus Severity Score to Predict Clinical Outcomes in Premature Neonates

**DOI:** 10.3390/jcdd9040114

**Published:** 2022-04-12

**Authors:** Krishna Kishore Umapathi, Brieann Muller, Cyndi Sosnowski, Aravind Thavamani, Joshua Murphy, Sawsan Awad, John W. Bokowski

**Affiliations:** 1 Department of Pediatrics, Division of Pediatric Cardiology, Rush University Medical Center, Chicago, IL 60612, USA; brieann_a_muller@rush.edu (B.M.); cyndi_r_sosnowski@rush.edu (C.S.); joshua_murphy@rush.edu (J.M.); sawsan_m_awad@rush.edu (S.A.); john_w_bokowski@rush.edu (J.W.B.); 2 Department of Pediatrics, Division of Pediatric Gastroenterology, UH Rainbow Babies Children’s Hospital, Case Western Reserve University, Cleveland, OH 44106, USA; aravind.thavamani@uhhospitals.org

**Keywords:** patent ductus arteriosus, prematurity, chronic lung disease, severity score

## Abstract

**Background:** Patent Ductus Arteriosus (PDA) in premature neonates has been associated with comorbidities including chronic lung disease (CLD), and death. However, the treatment of PDA remains controversial. There have been several echocardiographic variables previously used to determine the hemodynamic significance of PDA but their utility in early prediction of clinical outcomes is not well studied. **Objective:** The objective of our study was to evaluate the use of a severity scoring system incorporating markers of systemic under perfusion, pulmonary over perfusion and left ventricular (LV) function in predicting clinical outcomes in premature neonates. **Methods:** It is a single center prospective observational study involving newborns < 32 weeks’ gestation. An echocardiogram was done within seven days of life to measure variables previously known to predict severity of shunting in PDA including pulmonary perfusion index (PPI). Predictors of CLD/death were identified using multivariate logistic regression. A severity score was derived and its ability to predict clinical outcomes was tested using a receiver operating characteristic curve. **Results:** We studied 98 infants with a mean (SD) gestation of 28.9 ± 1.91 weeks and birth weight of 1228.06 ± 318.94 g, respectively. We identified five echocardiographic variables along with gestational age that was independently associated with the outcome variable (PPI, LV output, Superior Mesenteric Artery [SMA] Velocity Time Integral [VTI], Peak diastolic flow velocity in Pulmonary Vein [PV Vd], and reversal of flow in diastole in descending aorta [DFR]). The range of severity score was 0 (low risk) to 12 (high risk). A higher score was associated with the primary outcome variable of CLD/death (7.5 [1.2] vs. 3.6 [1.5], *p* < 0.001). Our severity score had an area under the curve of 0.97 (95% CI 0.93–0.99, *p* < 0.001) for predicting CLD/death. **Conclusion:** Our new PDA severity score of 5.5 has a sensitivity and specificity of 94% and 93%, and positive and negative predictive values of 94% and 93%, respectively.

## 1. Introduction

Despite widespread discussion on what constitutes “hemodynamic significance” in a neonate with patent ductus arteriosus (PDA), the ambiguity still remains, with no consensus definition reached [1]. Many randomized control trials have found no role in the treatment of PDA for prevention of mid-term and long-term outcomes including intraventricular hemorrhage (IVH), chronic lung disease (CLD), necrotizing enterocolitis (NEC), neurobehavioral problems and mortality [2,3,4,5,6,7,8,9]. Despite these findings, physiologically PDA can lead to systemic under perfusion and pulmonary over circulation when the shunt volume across the PDA is high enough. However, so far there has been no demonstrated direct echocardiographic marker that determines the shunt volume across PDA directly with accuracy. Hence, surrogates of PDA shunt volume have been used to assess the presence of pulmonary over circulation (PDA diameter, left atrial size and aortic root diameter ratio [LA to AO ratio], and/or systemic under perfusion (decreased flow in the celiac artery and superior mesenteric artery [SMA], percentage of diastolic flow reversal in descending aorta). There are additional clinical and echocardiographic variables used to diagnose hemodynamically significant PDA (hsPDA) including gestational age and left ventricular (LV) diastolic function, but there is a lot of heterogeneity among studies with overt importance on PDA diameter [10,11].

To our knowledge, no study so far has directly assessed the flow in the branched pulmonary arteries as a surrogate measure of detecting hsPDA. We sought to derive a PDA severity score (PDAss) incorporating various echocardiographic and clinical variables to assist in diagnosing hsPDA. Although there are multiple scoring systems using echocardiographic variables, the recent scoring system by El-Khuffash et al. in a multi-center prospective study predicted CLD/death as a combined outcome in newborns < 29 weeks of gestation [12]. The score is a composite of four echocardiographic variables (PDA diameter, maximum velocity across PDA shunt, LV output, LV diastolic function) ranging from 0–13 with a cut off >5 giving a sensitivity and specificity of 92% and 87% respectively in predicting the combined outcome of CLD/death. We sought to be more inclusive and include all premature newborns < 32 weeks’ gestation and derive an unique model that directly measures pulmonary blood flow. By doing so, we sought we can more accurately predict CLD since excessive pulmonary blood flow is considered a key factor behind the development or exacerbation of CLD in premature newborns.

Current practice across NICUs in the United States is to use echocardiography as the standard tool for assessment of hemodynamic significance to supplement the limited ability of clinical and laboratory data. Therefore, having a robust technique to measure reliable variables that help in determining the flow dynamics of a PDA shunt is essential to inform early decision-making and assess the need for possible pharmacological treatment or device closure. We hypothesize that our technique of incorporating variables into a PDAss that directly assess flow across the PDA into the pulmonary arteries will predict our primary composite outcome of CLD/death before discharge with reasonable accuracy.

## 2. Materials and Methods

This is a prospective single center observational study conducted in the tertiary neonatal intensive care unit at Rush University Medical Center. We included all neonates admitted with a gestational age ≤ 32 weeks. Institutional Review Board approval was obtained. The presence of major congenital structural anomalies, pulmonary hypertension, and cardiac lesions other than PDA were excluded from the study except for patent foramen ovale (PFO). All echocardiograms were done within the first week of life, and at the time of the echocardiogram, none of the study subjects were medically treated for PDA. Treatment for PDA received after the echocardiogram, management of hypotension or pulmonary edema, if present, with fluids and inotropes or diuretics, respectively, was dependent on the discretion of the attending neonatologist who was blinded to the derived PDA score.

### 2.1. Clinical Data

Antenatal and postnatal data collected include gestational age and birthweight, gender, mode of delivery, 5 min APGAR score, cord pH, magnesium sulphate administration, antenatal steroids, chorioamnionitis, preeclampsia, chronological age in hours, vitals (heart rate, systolic and diastolic blood pressure), fluid intake (mL/kg/day), respiratory parameters including oxygen saturation, pH, mean airway pressure, fraction of inspired oxygen (FiO_2_), mode of respiratory support (invasive or noninvasive) at the time of the scan, medications (furosemide, inotropes, postnatal steroids) used after the echocardiogram during the hospital stay, clinical outcomes (culture positive sepsis, periventricular leukomalacia, intraventricular hemorrhage, necrotizing enterocolitis, chronic lung disease, death before discharge), and finally mode of closure of PDA (spontaneous, medical, interventional-transcatheter and surgical ligation). Periventricular leukomalacia was diagnosed when evidence of white matter degeneration was noted on cranial ultrasound or magnetic resonance imaging. Intraventricular hemorrhage (only Grade III or IV) was diagnosed by cranial ultrasound using Papile classification system [13]. Necrotizing enterocolitis was diagnosed with clinical suspicion plus evidence of pneumatosis intestinalis on the abdominal radiogram. Chronic lung disease was defined by the requirement of oxygen at 36 weeks of gestation.

### 2.2. Echocardiographic Data

The median (IQR) time period of performance of the echocardiography was 54 h (39–70). The scan was performed using the EPIQ CVx and EPIQ 7 Ultrasound systems (Philips Healthcare, a division of Philips North America LLC 22100 Bothell Everett Highway, Bothell, Washington 98041) by an experienced sonographer using a standardized protocol in accordance with recent guidelines published by the American Society of Echocardiography [14]. All images were digitally stored for further analysis and the additional measurements were extracted from the offline archive using Merge Cardio Software (IBM Watson Health, Cambridge, MA, USA) which serves as a secure storage platform for performed echocardiograms. Care was taken to rule out any congenital heart disease other than a PDA or a PFO and, if present, immediately communicated to the attending neonatologist.

Echocardiographic variables included surrogate markers of PDA characteristics, pulmonary over circulation and systemic under perfusion, and LV function: Left Pulmonary Artery (LPA) diameter (mm), LPA systolic and diastolic Velocity Time Integral (VTI) in cm, LPA flow (L/min/m^2^), Right Pulmonary Artery (RPA) diameter (mm), RPA systolic and diastolic VTI (cm), RPA flow (L/min/m^2^), PDA diameter (mm), PDA velocity (m/s), mitral inflow velocities E wave (m/s), A wave (m/s) and E/A ratio, peak diastolic flow velocity (PV Vd) in pulmonary vein (m/s), descending aorta flow (DFR) reversal (%), systolic and diastolic flow VTI in SMA and celiac artery (cm), left ventricular output (LVO in mL/kg/min), ratio of left atrial size to aortic root diameter (LA to AO ratio).

The PDA diameter was measured using a two-dimensional echocardiogram at the narrowest portion of the PDA. The maximum shunt velocity and flow across the PDA, LPA and RPA [Vmax] was measured with highest Nyquist limit needed for laminar flow. Tissue Doppler imaging (TDI) of the apical four-chamber view was used for LV early diastolic (e’), and late diastolic (a’) velocities (m/s) measured using a pulsed wave Doppler at the level of the lateral mitral valve annulus. If the e’ and a’ waves were fused, the visualized single wave was measured as the a’ wave. Flow (L/min/m^2^) in the pulmonary arteries was measured as follows: the branched pulmonary artery diameter (d) was measured 5 mm from the level of the bifurcation from the Main Pulmonary Artery (MPA) using the short parasternal axis view. Cross sectional area (CSA) of the branched pulmonary arteries were calculated from the diameter. The VTI of LPA and RPA were measured using the pulsed wave Doppler in the same view. The Doppler gate cursor was aligned to make sure it is parallel to the direction of flow in the branches. We did not use any angle correction and an average of 3 simultaneous Doppler wave forms were captured to be utilized for calculation of VTI. Pulmonary perfusion index (PPI) measuring flow in each of the pulmonary artery branches was then calculated using the following equation and then averaged to give a single value:

Pulmonary Perfusion Index (L/min/m^2^) = (CSA × VTI × heart rate) ÷ Body Surface Area. All measurements were performed by an experienced cardiac sonographer blinded to the clinical data. The primary outcome of the study was a composite of CLD/death before discharge. Based on the ability of the El-Khuffash score to predict CLD/death, we estimated that for our score to have at least a 92% area under the curve with a 95% confidence level and a confidence interval of 0.125 with 30% combined prevalence of our composite primary outcome, the expected total sample size was 88 [15].

### 2.3. Statistical Analysis

Our cohort was divided into two groups based on the presence of primary composite outcome of CLD/death. Categorical variables were expressed as *n* (%) and continuous variables as mean (SD) or median (IQR) when appropriate. Categorical and continuous variables will be compared by χ2/Fischer exact and Student’s *t*-test/Mann Whitney test as appropriate, respectively after testing for normality using the Shapiro-Wilk test. Univariate analysis was performed to identify the echocardiographic variables associated with primary outcome (Table S1). A *p* value of <0.05 was considered to be statistically significant. All statistical analysis were performed using IBM SPSS Statistics for Macintosh, v26.0 (IBM Corp, Armonk, NY, USA)

### 2.4. Developing the PDAss

We used the echocardiography variables obtained within one week of birth since it can help with early decision-making regarding treatment and also the clinical effects of a significant PDA shunt tend to occur after the first week of life. All echocardiographic parameters suggestive of either pulmonary over perfusion or systemic hypo perfusion along with measurements of LV diastolic function were included in the multivariate logistic regression analysis to derive the PDAss. Clinical variables included in the model include gestational age (in the format of weeks and days) which was decided a priori since it is an important known predictor of primary outcome. Collinearity diagnostics was done to remove variables that were highly correlated with each other (VIF > 2.5). A *p* value > 0.05 was considered non predictive and those variables were removed from the final model. Five echocardiography variables were included in the final model: PPI (L/min/m^2^), LVO (mL/kg/min), PV Vd (m/s), SMA VTI (cm), and DFR (%). The variables in the final model were again tested for collinearity. The variance inflation factor for all the variables was found to be less than 1.5.

A weighted scoring system based on the beta coefficients of the significant predictors was used to derive the PDAss. The following equation was used to calculate the risk score for each infant:

(Gestational Age × −0.359) + (PPI in L/min/m^2^ × 0.418) + (LVO in mL/kg/min × 0.001) + (SMA VTI in cm × −0.041) + (PV Vd in m/s × 0.434) + (DFR in % × 0.150) + 11 (constant)

The score ranges from 0 (low risk) to 12 (high risk). The predicted probability for each infant to develop CLD/death was also derived using the same model. We then compared the predictive accuracy of PDAss against PDA diameter, LA:Ao ratio and DFR independently.

## 3. Results

One hundred and twenty-four patients with PDA detected during the first week of life were enrolled during the study period and 98 infants met the inclusion criteria (pulmonary hypertension [*n* = 20], major congenital anomalies [*n* = 3], inadequate data [*n* = 3] were excluded). The mean gestational age and birth weight of the study cohort was 28.9 ± 1.91 weeks and 1228.06 ± 318.94 g, respectively. Thirty-four infants met criteria for the primary outcome of CLD/death (death [*n* = 2]). The cause of death was severe respiratory failure in both neonates, one of which also had culture proven gram-negative sepsis. Table 1 demonstrates the comparison of demographic information, perinatal data and clinical characteristics among both the groups. Infants with the primary outcome had significantly lower gestation age and birth weight. Additionally, there was a higher incidence of pre-eclampsia and comparatively lower incidence of the administration of antenatal steroids to the mother before delivery in the CLD/death group. Among clinical characteristics, lower pH, slightly higher mean airway pressure, upper range of fraction of inspired oxygen, and marginally lower blood pressures were noted in the CLD/death cohort. Although statistically significant, the clinical relevance of these differences was low. Table 2 illustrates the differences in other clinically relevant clinical outcomes among both of the groups. Longer days of invasive ventilation, increased use of postnatal steroids, furosemide and inotropes were notable in the CLD/death group. The incidence of severe IVH and culture positive sepsis was higher in the CLD/death cohort. Other outcomes including NEC (higher incidence but not statistically significant), periventricular leukomalacia (PVL) and pulmonary hemorrhage (low *n*) were compared between the groups. Due to the low prevalence of PVL and procedural closure of PDA, calculations were not computed.

### 3.1. Comparison of Echocardiography Variables Using Univariate Analysis

**Figure 1:**
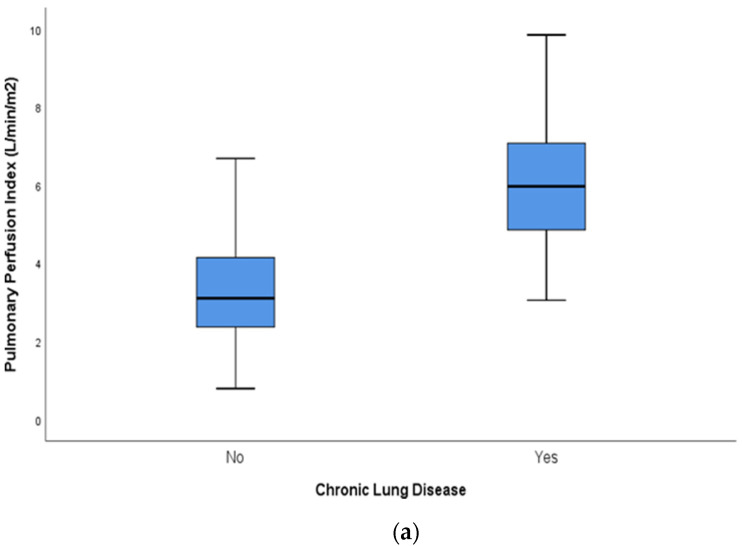

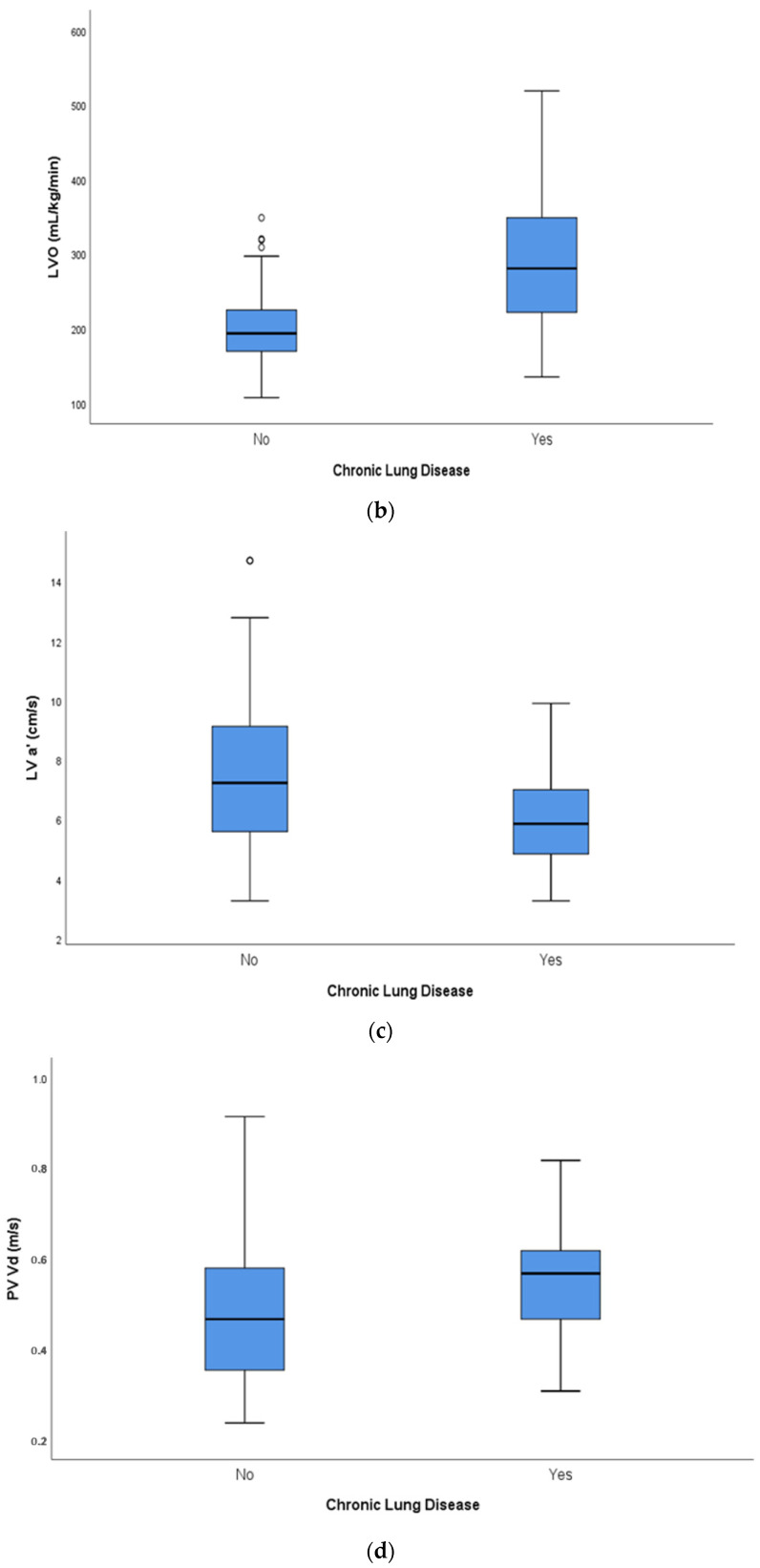

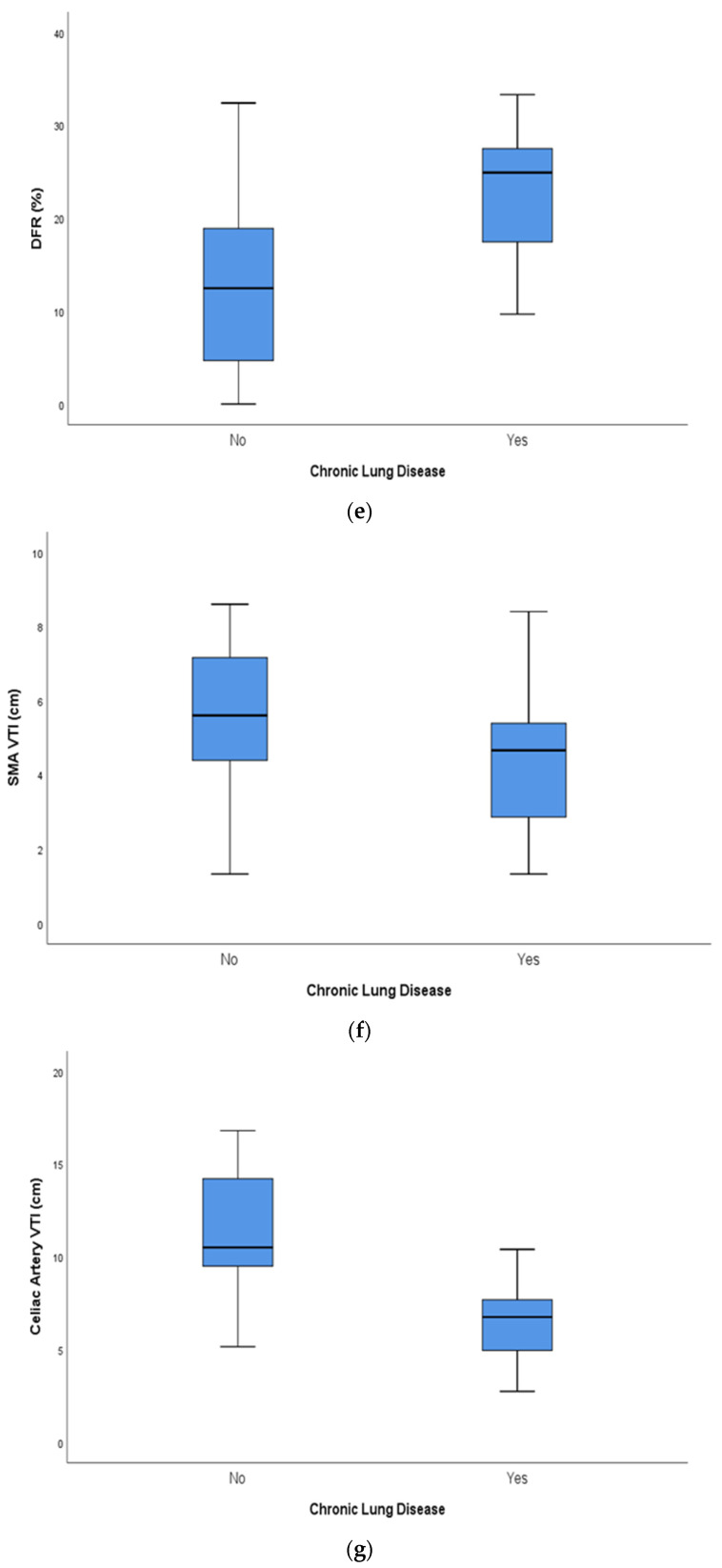

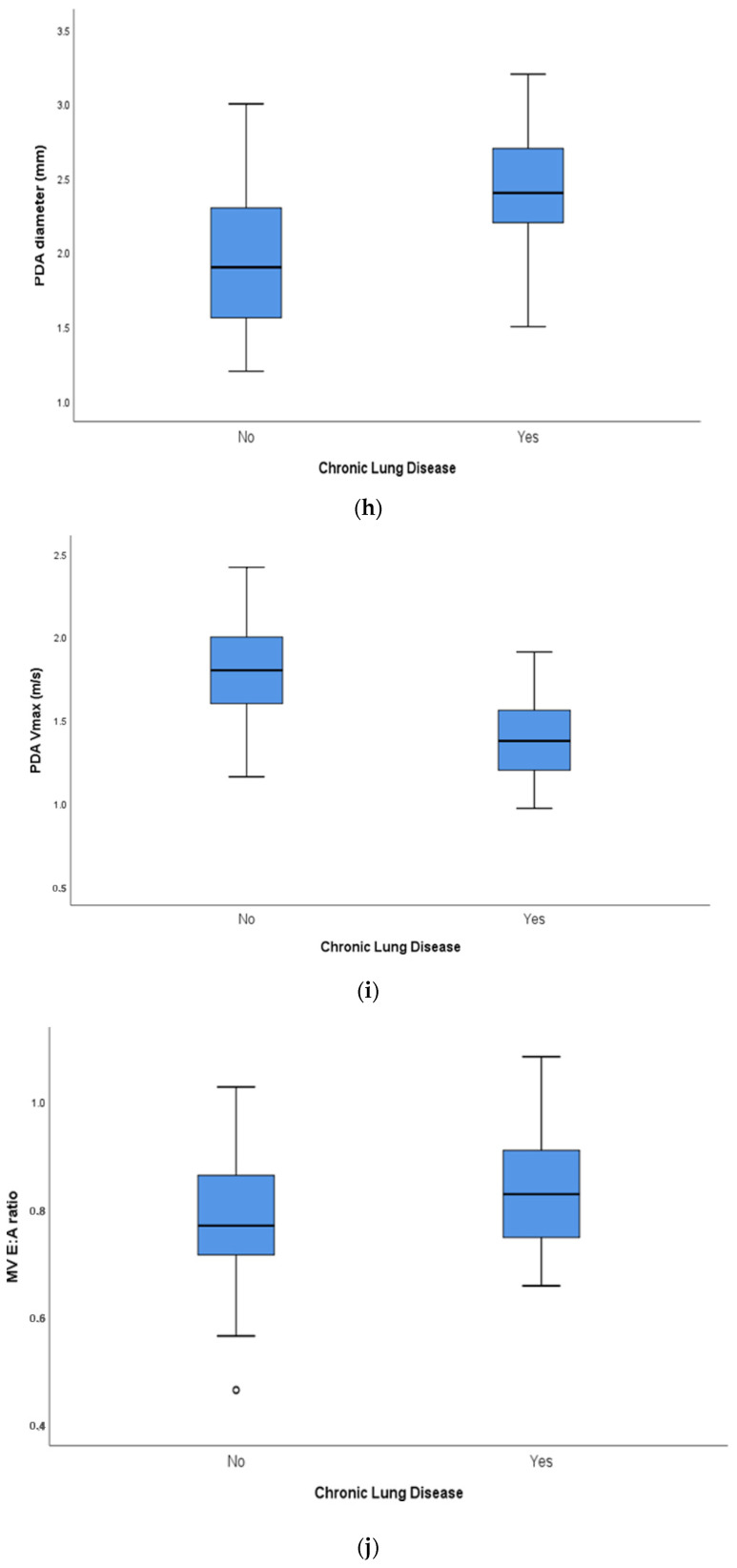

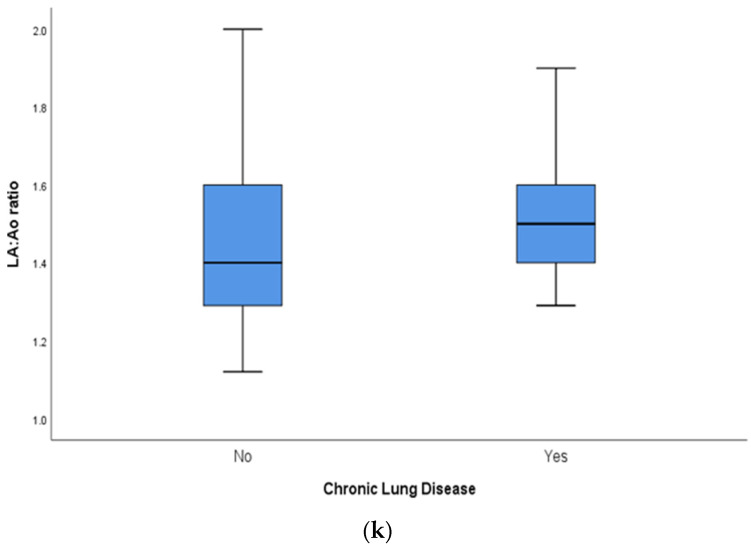
Comparison of echocardiography measurements in infants with and without CLD/death. (**a**): PPI; (**b**): LV Output; (**c**): LV a’; (**d**): Pulmonary Vein diastolic flow velocity; (**e**): Flow reversal in descending aorta; (**f**): SMA VTI; (**g**): Celiac artery VTI; (**h**); PDA diameter; (**i**): PDA Vmax; (**j**): Mitral Valve E/A ratio; (**k**): LA:AO ratio. The unfilled dots are shown in Figure 1b,c,j to indicate outliers.

Figure 1 illustrates the different boxplots that show the performance of all individual echocardiography variables measured for eligibility to be included in devising the PDAss. PPI was significantly higher in the CLD/death group (*p* < 0.001). Similarly, LV output (*p* = 0.01), pulmonary vein diastolic flow velocity (*p* = 0.05), SMA VTI (*p* = 0.03), and diastolic flow reversal in the descending aorta (*p* = 0.01) were all significantly higher when they met criteria for the primary outcome. PDA diameter, PDA Vmax, LV a’ measured by TDI, LA:Ao ratio and mitral valve E/A ratio were not statistically significant.

### 3.2. PDAss

**Figure 2:**
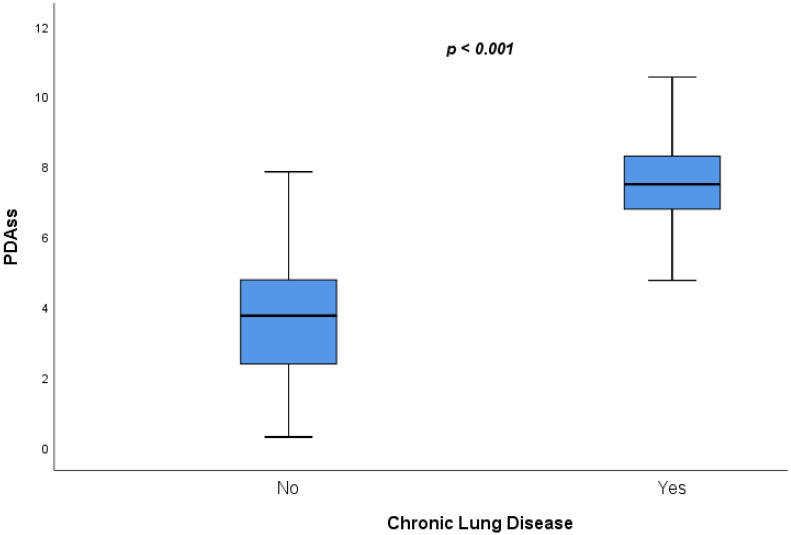
Difference in PDAss between infants with and without CLD/death.

**Figure 3:**
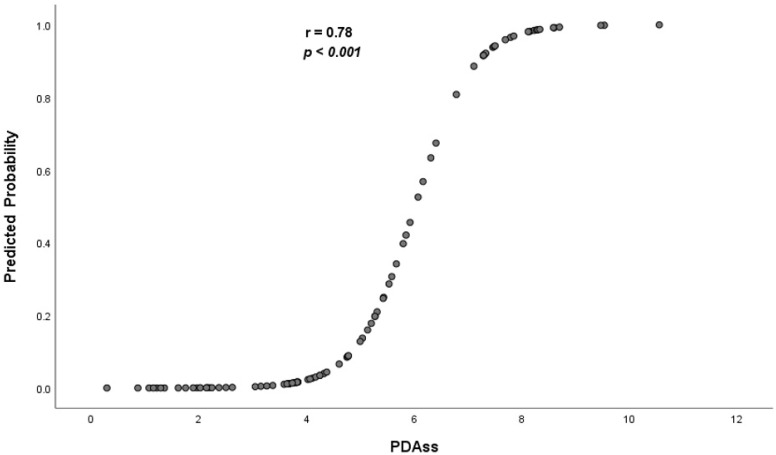
Relationship between PDAss and predicted probability of CLD/death of the entire cohort.

Six variables [gestational age and five echocardiography variables—PPI (L/min/m^2^), LVO (mL/kg/min), SMA VTI (cm), PV Vd (m/s), and DFR (%)] were included in the final multivariate logistic regression model which was then used to devise the severity score. Table 3 demonstrates the unstandardized β coefficients of the predictor variables which denote the relative importance and significance of each independent variable. Subsequently, a PDAss was derived ranging from 0 (lowest risk) to 12 (highest risk). The mean risk score of the entire cohort was 4.99 ± 2.35, while the infants with CLD/death endpoint had a higher score (7.5 [1.2] vs. 3.6 [1.5], *p* < 0.001) compared to those who did not have CLD/death (Figure 2). A strong correlation was demonstrated between the predicted probability of developing the primary outcome which was derived using the model and the PDAss (Figure 3).

The unstandardized beta coefficients were used to devise the risk score (see Methodology section for details). Negative β coefficients indicate that higher variable values are associated with a decrease in the risk of developing the outcome. Positive β coefficients indicate that higher variable values are associated with an increase in the risk of developing the disease.

**Figure 4:**
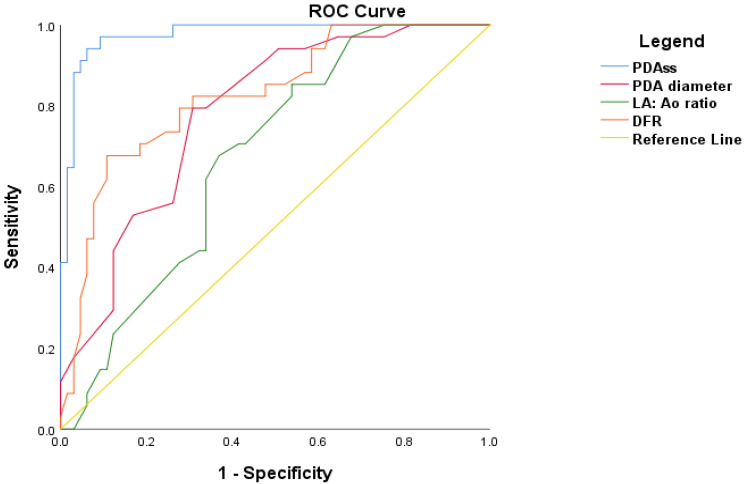
Receiver operating characteristics curve of the ability of PDAss to predict CLD/death.

A receiver operating characteristics curve was constructed to probe the predictive ability of the severity score to predict CLD/death in the study population. PDAss yielded an area under the curve (AUC) of 0.97 (95% CI 0.93–0.99, *p* < 0.001) for predicting CLD/death. When compared with individual parameters including PDA diameter (AUC), LA:Ao ratio (AUC) and DFR (AUC), which are traditionally used to demonstrate hemodynamic significance, our score fared better (Figure 4). A cut-off of 5.5 on the PDA severity score had a sensitivity and specificity of 94% and 93%, respectively, and positive and negative predictive values of 94% and 93%, respectively. In our cohort, 36 infants (37%) had a score higher than 5.5.

The relationship between the PDAss and the primary outcome was further probed by controlling for other clinical variables listed in Table 2 that were associated with CLD/death on univariate analysis. After controlling for severe IVH, sepsis, postnatal steroid use, furosemide use, and inotrope support, PDAss remained significant [aOR 2.76 (95% CI 1.89–3.52), *p* < 0.001]. Finally, PDAss was compared between infants with and without NEC, showing a higher score in those with as compared to those without NEC (6.5 ± 2.7 vs. 3.8 ± 1.7, *p* = 0.03). The AUC for NEC predictive ability of the score was 0.76 (95% CI [0.59–0.94], *p* = 0.02).

## 4. Discussion

Our study provides a novel echocardiography-based severity score with high accuracy in predicting CLD or death. The high reliability of the score is secondary to the use of a combination of markers of systemic under perfusion and pulmonary over perfusion rather than individual markers alone. Also, the differences in these parameters, specifically the pulmonary perfusion index and diastolic velocity of pulmonary veins, both markers of pulmonary over circulation, seems to be highly predictive of CLD or death and can be demonstrated in the first week of life. Along with other markers including gestational age and echocardiography parameters of the left ventricular output, flow in the superior mesenteric artery, and reversal of flow in the descending aorta, the derived score can play a vital role in predicting clinical outcome of CLD or death in infants < 32 weeks.

Early treatment of a patent ductus arteriosus (PDA) in preterm infants has been controversial. The left to right shunt of blood across the PDA can lead to both pulmonary over circulation and also compromises systemic perfusion [16]. Previous studies have demonstrated the association of a patent ductus with important clinical outcomes including necrotizing enterocolitis (NEC), intraventricular hemorrhage (IVH), bronchopulmonary dysplasia, death, and abnormal neurodevelopment in preterm infants [3,4,5,6,7,17]. However, randomized control trials have failed to demonstrate to proceed further in demonstrating causality related to PDA and the above-mentioned outcomes [8,18,19,20]. This could be related to an important limitation in these studies—they failed to specifically study the early hemodynamic impact of the PDA before initiating treatment. This can have a major impact in studying the effect of the treatment measures for PDA on clinical outcomes both in the short-term and long-term periods.

A patent ductus in the early life of a preterm infant can be physiologic, sometimes beneficial (in the presence of pulmonary hypertension), or pathologic (the presence of pulmonary congestion and systemic under perfusion). Hence, it becomes very difficult to assess the hemodynamic aspects of a PDA only by evidence of clinical signs, and the potential benefits of using an echocardiogram plays an important role in not only the diagnosis, but also in defining the role of a PDA in the development of important clinical outcomes, thereby helping determine the need for early treatment [21].

There have been several variables, both clinical and echocardiographic, that have been used to try defining what constitutes a “hemodynamically significant” PDA [11]. However, there is no clear justification or validation of these in the prediction of clinical outcomes [22,23]. Instead, the cut off index of these echocardiography-based variables were decided based on whether they are associated with clinical features of a PDA or whether the infant received treatment or not [24].

Prior to the routine use of echocardiography to confirm the presence of a PDA, physicians were reliant on clinical signs associated with the diagnosis of a PDA (continuous machinery like murmur, widened pulse pressure, bounding peripheral pulses, and increased precordial activity). However, it is important to know that the echocardiographic diagnosis of a significant PDA precedes the development of these clinical signs by at least two days [24]. Furthermore, it was found that there is a poor correlation between the presence of a PDA and these clinical signs, as demonstrated by echo in the first week of life [24,25]. Bounding pulses and a murmur may be absent in up to 1/5th of infants with a patent ductus [26]. Therefore, echocardiography still remains as the gold standard method for diagnosis of PDA [24].

There is evidence suggesting that a PDA associated with significant pulmonary over perfusion along with left ventricular volume overload and systemic under perfusion will benefit from treatment [22]. In addition, these markers may be able to identify poor neurodevelopmental outcomes associated with a PDA [5]. When institutes adopt a conservative approach in the management of PDA as opposed to an early targeted approach [27,28], a four- to seven-fold increase in mortality has been reported [6]. In premature newborn baboons, the presence of PDA with increased flow to the pulmonary circuit lead to impaired lung function and the arrest of alveolar development and a decrease in surface area with prevention of these outcomes demonstrated by pharmacological closure [29].

It is not possible to directly measure PDA shunt volume; however, surrogate echocardiography markers of pulmonary over-circulation and systemic hypoperfusion can be used to assess the magnitude of the shunt volume indirectly. Hence, a PDA severity score that incorporates a combination of these variables might be able to predict clinical outcomes with more accuracy. Sehgal et al devised an echo-based PDA severity score for preterm infants with measurements recorded at the time of PDA treatment (at approximately seven days of life) that could be associated with CLD [30]. However, application of a staging system for severity of hemodynamic impact of a PDA at an earlier timepoint can help with more targeted treatment of PDA earlier in life. Sellmer et al. [31] demonstrated that in infants born at less than 28 weeks of gestation, the presence of a large PDA (based on PDA diameter) on day three of age are associated with increased odds of IVH, CLD, and mortality, although causality could not be directly extrapolated. Schena et al [32], based on the PDA severity score developed by McNamara and Sehgal, illustrated that the length of exposure to a more severe PDA is associated with adverse outcomes. However, when PDA severity was accounted for, PDA ligation was not associated with CLD. Although these studies do not prove a cause and effect relationship between the presence of a PDA and development of CLD, there is increasing evidence that this might be true in a select population of infants with PDA who have an increased shunt volume.

Due to the need for a comprehensive score that can predict and discriminate the severity of PDA using echocardiographic measurements documented within 48 h of life [33], we sought to develop a PDA severity score and associate it with comorbidities that have traditionally been linked with PDA in premature neonates. Since gestational age plays a vital role in the development of these comorbidities, it was included a priori in the development of our score. Other components of the score should include a combination of markers of pulmonary over circulation and systemic under perfusion. We also assessed for markers of LV diastolic function, as it plays a role when there is increased blood volume on the left side of the heart (increased preload). However, due to high collinearity with other variables, they were not included in the final model. Celiac artery VTI, a marker of systemic perfusion, which was significantly lower in the CLD/death cohort, was also not included in the model due to high collinearity with other markers of systemic under perfusion. The relative influence of each variable can also be inferred from our study, as shown in Table 3, with pulmonary vein diastolic velocity and PPI having the biggest influence (high unstandardized beta coefficients) followed by gestational age, whereas LVO and SMA VTI had relatively lower significance. This could be explained by the fact that markers of pulmonary over circulation play a more important role in development of CLD than do surrogates of systemic under perfusion. More importantly, PDA diameter was not significantly different among both groups to be included in the model, reiterating the knowledge that it is the pressure difference across the duct and not the size that determines pulmonary overflow.

Although various clinical outcomes including NEC, IVH, invasive ventilation and sepsis can influence our primary outcome, they were not used in devising the PDAss due to the fact that most of them are diagnosed later than the first week of life and their early predictive ability becomes questionable and it is hence not ideal to include them in the model. Other cardiorespiratory characteristics like ventilatory settings, blood pressure and oxygen requirement were highly correlated with gestational age and therefore not considered for devising the score.

Although CLD and death were mutually exclusive outcomes, we developed a composite, as the number of deaths in our cohort was low, making it difficult to assess the significance of the association. Similarly, we also studied the accuracy of the score in predicting NEC independently, with an AUC of 0.76. The cut off yield of 5.5 had the best sensitivity and specificity. More importantly, the negative and positive predictive values of this cut off was relatively high as well, implying the generalizability to other populations. Hence, our score can be used with this cut off to differentiate severity in further prospective studies for external validation and also have a potential use in randomized clinical trials to determine selection for early treatment.

### Limitations

Although it was a prospective study, there is always a possibility of selection bias due to its observational nature given that it was performed at a single tertiary care unit. There was a relatively low incidence of IVH and NEC in the study cohort. This may be due to the non-selective inclusion of premature infants less than 32 weeks of age (as opposed to the use of extremely low weight and extremely premature infants) and the relatively high percentage of medical treatment of PDA at our center. In addition, although we had a standardized unit protocol for neonatal echocardiography in the assessment of PDA, the echocardiograms were performed by a single experienced sonographer and hence the reproducibility and inter observer variability of variables obtained live during active image acquisition and/or off-line analysis was not tested. However, the intra observer variability measured by the intra class correlation coefficient (ICC) for variables that were not routinely performed on transthoracic echocardiograms on all infants before the start of the study including VTI of the branched pulmonary arteries (ICC 0.94), and VTI of the celiac (ICC 0.82) and superior mesenteric arteries (ICC 0.87) showed excellent intra observer reliability. In addition, measurements of VTI of branched pulmonary arteries are not routinely performed by all sonographers or cardiologists and might depend on the performer’s expertise and the equipment available at disposal. The timing of the performance of an echocardiogram during the first week of life can have an effect on PDAss, although this is unlikely given they were PDA treatment naive premature newborns. CLD severity was not assessed and was included as a dichotomous variable [oxygen requirement at 36 weeks gestational age (GA) or at discharge (whichever comes first)] for ease of use in the regression model, and hence some milder forms might have been missed. Due to our low sample size, we were not able to assess the performance of the PDAss on other important clinical outcomes including pulmonary hemorrhage and IVH. Also, the effect of PDA ligation on CLD was also not assessed due to the same reasons. Importantly, our score cannot be generalized yet as it has not been externally validated in another cohort.

## 5. Conclusions

A cutoff score of our PDAss of 5.5 assessed within the first week of life on infants less than 32 weeks of gestation puts 37% of our cohort at high risk for adverse clinical outcomes, which might indicate the institution of early management of PDA. If our severity score is validated in further prospective multi-institutional studies, it can be used in conducting randomized control trials for early targeted treatment of PDA.

## Figures and Tables

**Table 1:** Demographic characteristics and clinical details among both groups.

Characteristics	CLD/Death (*n* = 34)	No CLD/Death (*n* = 64)	*p* Value
Gestation (wk.)	27.39 ± 1.44	29.74 ± 1.61	<0.001
Birth weight (g)	1038.65 ± 215.02	1317 ± 327.20	<0.001
Male	22 (64%)	37 (58%)	0.33
Vaginal delivery	21 (62%)	36 (56%)	0.42
Preeclampsia	9 (26%)	9 (14%)	0.04
Use of MgSO_4_	23 (68%)	40 (63%)	0.67
Chorioamnionitis	3 (9%)	5 (8%)	0.91
Cord pH	7.31 ± 0.12	7.33 ± 0.16	0.82
Antenatal Steroids			0.04
None	4 (12%)	9 (14%)	
1 dose	8 (24%)	19 (30%)	
2 doses	22 (64%)	36 (56%)	
Apgar score at 5 min	8 [6–9]	9 [7–9]	0.16
Clinical characteristics *			
pH	7.29 ± 0.11	7.31 ± 0.14	0.03
Mechanical ventilation	19 (56%)	27 (42%)	<0.001
Mean airway pressure (mmHg)	8 ± 2	7 ± 2	0.05
Inspired oxygen fraction † (%)	21 [21–67]	21 [21–44]	<0.001
Oxygen saturation (%)	95 ± 3	97 ± 2	0.03
Total fluid intake (mL/kg/day)	110 [100–130]	100 [90–120]	0.15
Systolic blood pressure (mmHg)	55 [50–60]	57 [51–65]	0.04
Diastolic blood pressure (mmHg)	29 [22–36]	32 [27–38]	0.02

MgSO_4_—Magnesium sulphate; Values are presented as *n* (%) and Mean ± SD or Median [IQR] as appropriate. † Presented as median [range]. * Clinical characteristics displayed were recorded at the time of the echocardiogram.

**Table 2:** Distribution of clinical outcomes and interventions among both groups.

	CLD/Death BeforeDischarge	No CLD/Death BeforeDischarge	*p* Value
NEC	5 (15%)	3 (5%)	0.07
Clinical suspicion	2	2	
Medical therapy	2	1	
Surgical therapy	1	0	
Days of mechanical ventilation	18 [6–34]	2 [0–5]	<0.001
Pulmonary hemorrhage	1	0	-
Furosemide	17	12	<0.001
Inotropes	14	7	<0.001
Postnatal steroids	12	2	<0.001
Culture-positive sepsis	3	1	<0.001
IVH (Grade 3 or 4)	2	1	0.004
PVL	1	0	-
PDA treatment (after 1 week of life)	20 (58%)	12 (19%)	<0.001
Ibuprofen	20 (58%)	12 (19%)	<0.001
Paracetamol	8 (24%)	4 (6%)	<0.001
Trans catheter Closure	3 (9%)	0	-
Surgical Ligation	1 (3%)	0	-

**Table 3:** Regression model used to derive PDAss.

Predictor Variable	Unstandardized β	Standardized β	*p* Value
Gestational Age	−0.359	−0.086	<0.01
Pulmonary Perfusion Index (L/min/m^2^)	0.418	0.506	<0.01
LVO (mL/kg/min)	0.001	0.187	0.01
SMA VTI (cm)	−0.041	−0.152	0.02
PV Vd (m/s)	0.434	0.151	0.02
DFR (%)	0.150	0.298	0.01

## Data Availability

The data presented in this study are available on request from the corresponding author.

## Supplementary Materials

The following are available online at https://www.mdpi.com/article/10.3390/jcdd9040114/s1, Table S1: Univariate Analysis involving gestational age and all echocardiography variables to predict Chronic Lung Disease.

## Abbreviations

AUC	Area Under the Curve
CLD	Chronic Lung Disease
EF	Ejection Fraction
DFR	Descending aorta Flow Reversal
GA	Gestational Age
hsPDA	hemodynamically significant Patent Ductus Arteriosus
ICC	Intraclass Correlation Coefficient
IVH	Intraventricular Hemorrhage
LA	Left Atrium
LPA	Left Pulmonary Artery
LV	Left Ventricle
LVO	Left Ventricular Output
MPA	Main Pulmonary Artery
NEC	Necrotizing Enterocolitis
PDA	Patent Ductus Arteriosus
PDAss	Patent Ductus Arteriosus severity score
PFO	Patent Foramen Ovale
PPI	Pulmonary Perfusion Index
PVL	Periventricular Leukomalacia
PV Vd	Peak diastolic flow velocity in Pulmonary Vein
RPA	Right Pulmonary Artery
SMA	Superior Mesenteric Artery
TDI	Tissue Doppler Index
Vmax	Maximum Velocity
VTI	Velocity Time Integral
