# Supplementary material for: A Novel Patent Ductus Arteriosus Severity Score to Predict Clinical Outcomes in Premature Neonates
Source: J Cardiovasc Dev Dis. 2022 Apr 12;9(4):114. doi: 10.3390/jcdd9040114 (PMC9033137; doi:10.3390/jcdd9040114)
Supplement: Supplementary file 1 [file jcdd-09-00114-s001.zip › Table S1 - Univariate Analysis.pdf]

Table S1. Univariate Analysis involving gestational age and all echocardiography variables to predict Chronic Lung Disease.

Predictor Variables	No CLD/Death	CLD/Death	P value
Gestational Age (wk)	29.74±1.61	27.39±1.44	<0.001
PPI (L/min/m²)	3.12±1.25	6.15±1.85	<0.001
PDA diameter	1.92±0.48	2.42±0.42	<0.001
PDA Vmax	1.78±0.33	1.37±0.26	0.002
LVO (mL/kg/min)	206.26±53.08	292.52±93.41	0.01
Celiac Artery VTI (cm)	11.30±3.00	6.50±1.69	<0.001
SMA VTI (cm)	5.61±1.67	4.37±1.72	0.002
LV a′ (cm/s)	7.57±2.85	6.28±1.74	0.07
PV Vd (m/s)	0.49±0.17	0.56±0.14	0.04
LA:Ao ratio	1.44±0.19	1.52±0.16	0.03
MV E:A ratio	0.79±0.12	0.83±0.11	0.09
DFR (%)	12.08±8.97	23.00±6.97	<0.001
